# Comparison of analgesia and akinesia between sub-Tenon’s capsule anesthesia and trans-Tenon’s capsule retrobulbar anesthesia in vitrectomy

**DOI:** 10.20407/fmj.2020-017

**Published:** 2020-11-13

**Authors:** Ryoko Nomura, Yoshiaki Shimada, Mitsuo Sugimoto, Atsuhiro Tanikawa, Tadashi Mizuguchi, Masayuki Horiguchi

**Affiliations:** Department of Ophthalmology, Fujita Health University, School of Medicine, Toyoake, Aichi, Japan

**Keywords:** Sub-Tenon’s capsule anesthesia, Trans-Tenon’s capsule retrobulbar anesthesia, Akinesia, Pain, Phacovitrectomy

## Abstract

**Objectives:**

We compared the effects of sub-Tenon’s capsule anesthesia (STA) and trans-Tenon’s capsule retrobulbar anesthesia (TTRBA) in 68 patients with epiretinal membrane.

**Methods:**

Either STA or TTRBA was induced with 3 mL of lidocaine (2%) before vitrectomy combined with phacoemulsification and aspiration (phacovitrectomy). Akinesia was evaluated by range of eye movement (ROEM) in upward, downward, nasal, and temporal directions at 4, 10, and 30 minutes after injection. Analgesia was evaluated with a visual analogue pain score, which ranged from 0 to 10.

**Results:**

The mean cumulative ROEMs were 1.44±1.02 corneal diameters (CDs) at 4 minutes, 0.55±0.76 CDs at 10 minutes, and 0.26±0.33 CDs at 30 minutes in patients who received STA; these values were 0.39±0.35 CDs at 4 minutes, 0.22±0.30 CDs at 10 minutes, and 0.13±0.29 CDs at 30 minutes in patients who received TTRBA. At both 4 and 10 minutes, the cumulative ROEMs in all directions, as well as the temporal ROEMs, were significantly larger in patients who received STA than in patients who received TTRBA. Pain scores did not significantly differ between groups at any time point.

**Conclusions:**

STA and TTRBA produced identical degrees of analgesia, but akinesia was slower in patients who received STA. TTRBA might be preferable for patients undergoing brief vitrectomy.

## Introduction

Analgesia and akinesia are important components of intraocular surgery, such that various techniques have been established for their implementation. In 1884, Knapp first reported the use of retrobulbar anesthesia (RBA) to achieve analgesia and akinesia;^1^ in 1936, Walter and Atkinson confirmed the feasibility of this technique.^2^ Subsequently, RBA has become a standard local anesthetic technique in intraocular surgery. The anesthetic is injected in the muscle cone of the orbital space, thereby blocking the ciliary ganglion and motor nerves. In 1986, Davis and Mendel^3^ and Bloomberg^4^ separately reported that peribulbar anesthesia constituted a safer technique than RBA; in peribulbar anesthesia, the anesthetic is injected outside the muscle cone and inside the orbital space, reaches inside the muscle cone several minutes after injection, and blocks the nerves. In 1992, Stevens^5^ developed sub-Tenon’s capsule anesthesia (STA), in which the anesthetic is injected between the sclera and Tenon’s capsule, thereby blocking sensory nerves that penetrate the sclera.^6–10^ Topical anesthesia has also been reported to cause fewer complications;^11^ however, it does not induce any degree of akinesia.

Currently, topical anesthesia is used in most cataract surgeries. However, STA or RBA is used in vitrectomy because the operation time is sometimes prolonged and the manipulation (e.g., peeling of the epiretinal or inner limiting membrane) is more delicate than the approach used in cataract surgery. Vitrectomy has been performed without premedication as outpatient surgery; in this approach, surgeons must carefully monitor the pain induced by induction of anesthesia. The original RBA technique includes transcutaneous insertion of a 22- or 23-gauge sharp needle (i.e., transcutaneous RBA), which causes severe pain. In contrast, the STA technique involves delivery of anesthetic through the conjunctiva; accordingly, the conjunctiva undergoes pretreatment with topical anesthesia, which leads to overall pain reduction. However, anterior leakage of the anesthetic in STA may cause edema to form over a large area of the conjunctiva (i.e., chemosis); thus, akinesia may be incomplete because the area below Tenon’s space is removed from the oculomotor and abducens nerves.

Sugimoto et al.^12^ combined the RBA and STA procedures into a so-called “trans-Tenon’s capsule retrobulbar anesthesia” (TTRBA).^12^ In TTRBA, the anesthetic can be injected inside the muscle cone through the conjunctiva; this involves less pain than that experienced during transcutaneous RBA. Notably, Sugimoto et al. showed no difference in the degrees of analgesia and akinesia between TTRBA and transcutaneous RBA.^12^

In the past, we performed transcutaneous RBA for vitreous surgery. This approach achieved suitable akinesia and analgesia, but caused considerable pain during injection; it also led to some serious complications, such as retrobulbar hemorrhage and eyeball perforation. Implementation of TTBRA eliminated the pain during injection and the risk of serious complications, thereby reducing stress for both the patient and the operator. In all of our facilities, we implemented TTRBA for all patients undergoing vitrectomy; however, in many other facilities, we implemented STA for patients undergoing other ocular surgeries that involve shorter operation times (e.g., cataract or glaucoma surgery). However, we have encountered instances in which patients exhibit insufficient akinesia and considerable intraoperative pain after use of STA, compared with TTRBA. We hypothesized that STA would not achieve adequate akinesia because the oculomotor nerves innervate muscles deep in the orbital space.13 In contrast, we hypothesized that TTRBA would achieve adequate akinesia because the cannula is placed below Tenon’s space and a long, sharp needle is inserted into the cannula to reach inside the muscle cone. Thus, the anesthetic is injected in the muscle cone where the ciliary ganglion resides; furthermore, the oculomotor nerve inserts into all muscles except the superior oblique muscles, which are innervated by the trochlear nerve. We presumed that there might be differences in terms of analgesia and akinesia effectiveness between STA and TTRBA, especially in short durations of 30 minutes or less.

Here, we compared TTRBA and STA in terms of analgesia and akinesia, in patients undergoing vitrectomy. To the best of our knowledge, there have been no reports regarding comparisons between retrobulbar anesthesia and STA in these patients.

## Methods

### Research ethics

This study was approved by the institutional review board of Fujita Health University (No. HM18-430). It was conducted in accordance with the principles of the Declaration of Helsinki, as well as the Guidelines for Medical and Health Research Involving Human Subjects established by the Japanese Ministry of Education, Culture, Sports, Science and Technology and the Japanese Ministry of Health, Labour and Welfare. Informed consent was obtained by the opt-out method. Opt-out information was presented as a poster at the Ophthalmology Outpatient Department.

### Patients

This retrospective study included 68 consecutive patients with idiopathic epiretinal membrane who underwent phacovitrectomy in our hospital from December 2018 to February 2019. STA or TTRBA was induced on a rotating basis, such that the method changed daily. Patients were excluded from this study if they had undergone previous eye surgery and/or if they had other diseases that might cause abnormalities in eyeball and eyelid movement (i.e. strabismus, oculomotor nerve paralysis, and senile ptosis).

### Sub-Tenon’s capsule anesthesia

**Figure 1:**
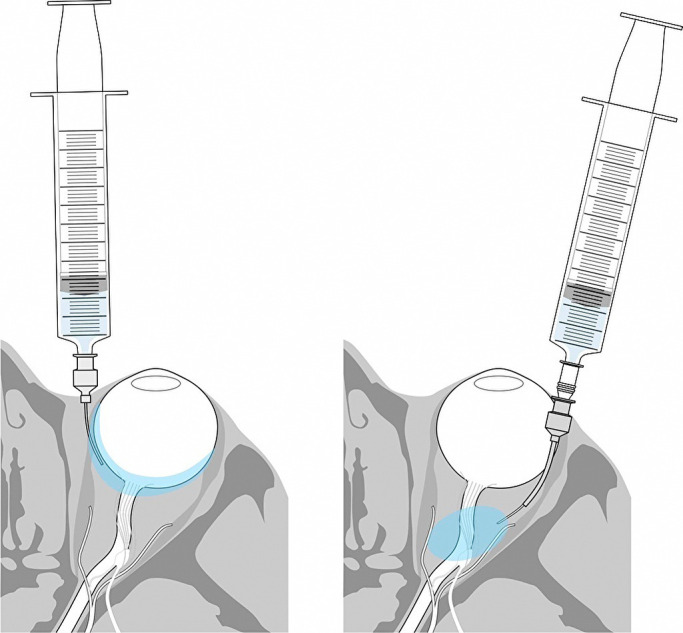
Procedures for induction of sub-Tenon’s capsule anesthesia (STA; left) and trans-Tenon’s capsule retrobulbar anesthesia (TTRBA; right). For STA, the sclera was exposed by an incision made in the conjunctiva and Tenon’s capsule in the inferonasal area. A 25-gauge dull needle designed for STA was inserted between the sclera and Tenon’s capsule; 3 mL of 2% lidocaine were injected below Tenon’s space. For TTRBA, the sclera was exposed in the inferotemporal area; a 23-gauge cannula designed for TTRBA was then inserted between the sclera and Tenon’s capsule. A 25-gauge sharp (50-mm) needle was then inserted in the cannula and 3 mL of 2% lidocaine were injected into the muscle cone.

Each patient’s ocular surface and eyelids were sterilized with povidone-iodine solution (0.05%); the upper and lower eyelids were then retracted using a speculum. The sclera was exposed by means of an incision in the conjunctiva and Tenon’s capsule in the inferonasal area. After sufficient hemostasis had been achieved by electrocoagulation, a 25-gauge dull needle designed for STA (Cannula Sub-Tenon Three-Port 25G; Inami, Tokyo, Japan) was inserted between the sclera and Tenon’s capsule (Figure 1, left panel), then used for injection of 3 mL of 2% lidocaine. No additional injection was administered, regardless of whether the lidocaine leaked anteriorly and caused chemosis.

### Trans-Tenon’s capsule retrobulbar anesthesia

In a manner similar to that of STA, the sclera was exposed in the inferotemporal area. A 23-gauge cannula designed for TTRBA was inserted between the sclera and Tenon’s capsule; a 25-gauge sharp (50-mm) needle (HS9970 Handaya, Tokyo, Japan) was then inserted in the cannula (Figure 1, right panel). When the needle was inserted, some resistance was encountered; the resistance disappeared after further insertion of the needle, which suggested that the needle had reached the inside of the muscle cone. Finally, 3 mL of 2% lidocaine were injected. No additional injection was administered for any patients who received STA or TTRBA.

### Surgical technique (phacovitrectomy)

After either STA or TTRBA had been induced, the surgeon delayed the start of surgery for 4 minutes. When surgery began, 25-gauge trocars were fixed on the sclera in the superotemporal, inferotemporal, and superonasal areas. A 3-mm corneal incision was made; the anterior chamber was then filled with viscoelastic material. The anterior capsule of the crystalline lens was removed; phacoemulsification and cortex aspiration were then completed.

A 25-gauge vitreous-cutter was used to remove as much of the vitreous as possible; the epiretinal and internal limiting membranes were then carefully peeled away. The intraocular lens was inserted in the lens capsule and viscoelastic material was removed. The three trocars were removed and the surgeon confirmed that no incisions exhibited leakage. STA, TTRBA, and surgery were all performed by a single surgeon who had substantial experience involving these three techniques.

### Evaluation methods

#### Akinesia of eye movement

Four, 10, and 30 minutes after injection, the ranges of eye movement (ROEMs) in four directions (up, down, nasal, and temporal) were measured under the operating microscope. The patient was asked to move the eye in each direction; the distances of corneal movement were measured in terms of corneal diameters (CDs). For example, if the distance the cornea moved was half the diameter of the cornea in the temporal direction, the temporal ROEM was recorded as 0.5 CDs.

#### Akinesia of the eyelid

At the end of surgery, the patient was asked to open the eye; eyelid movement was observed. No eyelid movement was scored as 0, slight movement was scored as 1, and free movement was scored as 2.

#### Analgesia

Intraoperative analgesia of the eyeball and its surroundings was evaluated with a visual analogue pain score.^6^ The patient was asked to indicate the number between 0 and 10 that best described pain intensity at the end of surgery. Zero represented no pain, while 10 represented the worst pain ever.

#### Complications

Chemosis and subconjunctival hemorrhage caused by the anesthetic technique were evaluated at the end of surgery.

### Statistics

Laterality and sex composition were compared between patients who received STA and those who received TTRBA, using the chi-squared test. Age was compared between groups using Student’s *t*-test. The Mann–Whitney *U* test was used to examine ROEM, eyelid movement score, and pain score, because the measured values of ROEM and eyelid movement and pain scores also did not exhibit normal distributions. ROEMs among directions in each group of patients were compared by using the Mann–Whitney *U* test with Bonferroni correction.

Since the Mann–Whitney *U* test t was performted six times in each direction, *p* values less than 0.0083 (0.05 divided by 6) were considered statistically significant.

## Results

Anesthesia was induced without serious complications, such as retrobulbar hemorrhage or eyeball perforation, and phacovitrectomy was successfully completed in all eyes. The results of comparisons between the two anesthetic techniques are shown in Table 1. Thirty-four patients (18 women, 14 men; mean age, 69.8±9.8 years) received STA; the other 34 (15 women, 19 men; mean age, 68.5±11.0 years) received TTRBA. The sex ratio and age did not significantly differ between the two groups of patients.

The mean cumulative ROEMs (based on data from all four directions) were 1.44±1.02 CDs at 4 minutes, 0.55±0.76 CDs at 10 minutes, and 0.26±0.33 CDs at 30 minutes in patients who received STA; these values were 0.39±0.35 CDs at 4 minutes, 0.22±0.30 CDs at 10 minutes, and 0.13±0.29 CDs at 30 minutes in patients who received TTRBA. The cumulative ROEMs were significantly larger in patients who received STA than in those who received TTRBA at 4 minutes (*P*=0.001, Mann–Whitney *U* test) and 10 minutes (*P*=0.021, Mann–Whitney *U* test). Among the individual directions, there were significant differences between the two groups in terms of upward ROEM (*P*=0.001), downward ROEM (*P*<0.0001), and temporal ROEM (*P*=0.011) at 4 minutes; upward ROEM (*P*<0.0001) and temporal ROEM (*P*=0.001) at 10 minutes; and temporal ROEM (*P*=0.048) at 30 minutes. Thus, STA was slower and less effective in achieving eye-movement akinesia; however, the surgeries were completed without any problem.

The mean eyelid movement scores were 1.12±0.95 in patients who received STA and 0.97±0.9 in patients who received TTRBA (*P*=0.441, *t*-test); the mean pain scores were 1.12±1.78 in patients who received STA and 0.53±10.3 in patients who received TTRBA (*P*=0.142, *t*-test). Thus, there were no significant differences in terms of eyelid akinesia or analgesia between the two groups. Subconjunctival hemorrhage was observed in two (5.9%) of the patients who received STA and in one (2.9%) of the patients who received TTRBA; chemosis was observed in two patients, both of whom had received STA.

The results of comparisons of directional differences of ROEMs in each technique are shown in Table 2. In STA recipients, significant directional differences were observed: 0.53±0.29 CDs in the temporal direction and 0.22±0.37 CDs in the nasal direction (*P*<0.0001, Mann–Whitney *U* test) at 4 minutes and 0.18±0.23 CDs in the temporal direction and 0.06±0.20 CDs in the nasal direction (*P*=0.0054, Mann–Whitney *U* test) at 10 minutes. In TTRBA recipients, no significant directional differences were observed.

## Discussion

At 9.5 mm posterior to the hind surface of the globe, the trochlear nerve, abducens nerve, and oculomotor nerve enter the muscle hila.^13^ In STA, 2% lidocaine is injected between the sclera and Tenon’s capsule; it reaches the posterior surface of the eyeball and blocks trigeminal nerves that penetrate the posterior sclera, which provides reasonable analgesia during intraocular surgery. However, STA may not achieve adequate akinesia in some patients because the oculomotor nerves innervate muscles deep in the orbital space.^13^ Conversely, in TTRBA, the cannula is placed below Tenon’s space; a long sharp needle is inserted into the cannula, then reaches inside the muscle cone. Therefore, the anesthetic is injected in the muscle cone, where the ciliary ganglion resides and the oculomotor nerve inserts into all muscles except the superior oblique muscles, which are innervated by the trochlear nerve. Sugimoto et al. previously reported that akinesia and analgesia produced by TTRBA were comparable to the outcomes of transcutaneous RBA.^12^

In this study, the cumulative ROEMs were significantly larger in patients who received STA than in those who received TTRBA at 4 and 10 minutes after injection; however, they did not significantly differ at 30 minutes after injection. ROEMs in each direction revealed differences between the two groups in upward, downward, and temporal directions at 4 minutes after injection; in upward and temporal directions at 10 minutes after injection; and in the temporal direction at 30 minutes after injection. Furthermore, ROEMs in the temporal direction (opposite from the injection) were significantly larger than ROEMs in the nasal direction in STA recipients at 4 and 10 minutes after injection, which suggested that anesthetic injected below Tenon’s space required more time to reach the oculomotor nerve.

In this study, based on standard clinical approaches for administration of anesthesia, STA was administered from the inferonasal direction, while TTRBA was administered from the inferotemporal direction. Importantly, this difference in the injection site might have affected the results. Although the effect of the injection site could be excluded by modifying the injection site, these injection sites were selected to enable evaluation of differences in anesthetic effects when standard clinical approaches were used.

In TTRBA recipients, because the anesthetic directly reaches the muscular cone, we presume that an anesthetic effect was achieved immediately, even at locations distal to the site of administration. In STA recipients, additional time may have been required to achieve an anesthetic effect in distal muscles because the anesthetic did not directly reach the muscle cone.

Furthermore, we examined whether STA and TTRBA caused different anesthesia effects depending on the direction of eye movement. In TTRBA recipients, no differences in anesthesia effects were detected with respect to eye movement direction at 4, 10, and 30 minutes after injection; although the injection site was on the lower temporal side, eye movement restriction was observed on the opposite side (i.e., nasal side). Conversely, in STA recipients, there were significant differences in superonasal, inferotemporal, and nasotemporal directions at 4 minutes after injection; there were significant differences in superonasal and nasotemporal directions at 10 minutes after injection. However, at 30 minutes after injection, there was no significant difference in any direction.

In STA, the administration site was located inferonasally; distal sites (i.e., superotemporal) were less likely to be restricted in movement, compared with nasal sites. Although it was not possible to exclude the effect of the difference in injection sites between STA and TTRBA, the difference in the effect of anesthesia depending on the direction of eye movement was presumed to be caused by the difference in anesthesia administration method.

All surgeries were successfully completed and no serious complications were observed. Moreover, the visual analogue pain scores of the two patient groups did not significantly differ. These results suggest that both anesthetic techniques can be used for vitrectomy. However, because the onset of oculomotor akinesia was delayed or because akinesia was less effective in STA recipients, TTRBA is preferable for vitrectomy procedures that can be completed in a short period of time. For example, vitrectomy for treatment of epiretinal membrane without concurrent cataract surgery is a shorter procedure than phacovitrectomy; membrane peeling may be initiated approximately 10 minutes after injection, at a time when akinesia is incomplete in STA recipients. The inner limiting membrane on the retina is 0.5 μm in thickness;^14^ peeling this membrane without damage to the retina requires eyeball immobility. Further development of surgical instruments in the near future may shorten operation time; TTRBA may then be preferable.

The limitations in this study involve the amount of anesthetic, degree of eyelid akinesia, and blockage of oblique muscles. Three milliliters of 2% lidocaine were used for both techniques because these are standard doses in Japan; if had we used larger volumes, the differences might have been smaller. Eyelid movement scores in the two patient groups were only measured at the end of surgery. However, the duration of eyelid akinesia may have differed between the two groups. To observe eyelid akinesia during surgery, the speculum must be removed, which considerably delays the completion of surgery; we did not have sufficient time in each procedure to perform this assessment. The upper and lower oblique muscles rotate the eye, but it is very difficult for the surgeon to measure eye rotation under the operating microscope; thus, we measured ROEMs only in horizontal and vertical directions. In addition, observer bias might have been present regarding measurements of eyeball and eyelid movement. Because the observer himself administered anesthesia, observer bias may have contributed to the reduction of measured values. To eliminate this, a masked observer could evaluate eye movements using surgical videos; however, the anesthesia method would be obvious because of conjunctival incision marks at the site of anesthetic injection, as well as hemostasis marks and the presence of conjunctival hemorrhage. Therefore, it would be difficult to fully mask an external observer.

In conclusion, both STA and TTRBA produced acceptable akinesia and analgesia for phacovitrectomy in our patients with epiretinal membrane; however, STA led to slower onset or less effective akinesia. Hence, the operation time must be considered during selection of the anesthetic technique.

## Figures and Tables

**Table1:** Comparisons of results between sub-Tenon’s capsule anesthesia and trans-Tenon’s capsule retrobulbar anesthesia

		STA	TTRBA	*P*
*n*		n=34	n=34	
Eye (right/left)		13/21	20/14	0.089^a^
Sex (female/male)		18/16	15/19	0.467^a^
Age (years; mean±standard deviation)		69.8±9.8	68.5±11.0	0.602^b^
Range of eye movement (ROEM), in corneal diameters (CDs)				
4-min	Total	1.44±1.02	0.39±0.35	**0.001** ^c^
	Upward	0.39±0.25	0.10±0.12	**0.001** ^c^
	Downward	0.29±0.31	0.10±0.14	**0.011** ^c^
	Nasal	0.22±0.37	0.10±0.17	0.516^c^
	Temporal	0.53±0.29	0.10±0.12	**0.001** ^c^
10-min	Total	0.55±0.76	0.22±0.30	**0.021** ^c^
	Upward	0.16±0.21	0.06±0.10	**0.029** ^c^
	Downward	0.15±0.22	0.06±0.11	0.116^c^
	Nasal	0.06±0.20	0.07±0.15	0.562^c^
	Temporal	0.18±0.23	0.03±0.05	**0.001** ^c^
30-min	Total	0.26±0.33	0.13±0.29	0.051^c^
	Upward	0.10±0.14	0.04±0.10	0.131^c^
	Downward	0.06±0.09	0.03±0.10	0.187^c^
	Nasal	0.01±0.06	0.05±0.13	0.522^c^
	Temporal	0.10±0.16	0.01±0.03	**0.048** ^c^
Lid movement score (range, 0–2)		1.12±0.95	0.97±0.94	0.441^c^
Pain score (range, 0–10)		1.12±1.78	0.53±1.03	0.142^c^
Subconjunctival hemorrhage		2 (5.9%)	1 (2.9%)	

^a^ Chi-squared test; ^b^ Student’s t-test; ^c^ Mann–Whitney *U* test. STA, sub-Tenon’s capsule anesthesia; TTRBA, trans-Tenon’s capsule retrobulbar anesthesia. *P* values in boldface are statistically significant.

**Table2:** Comparisons of directional differences of ROEM in sub-Tenon’s capsule anesthesia and trans-Tenon’s capsule retrobulbar anesthesia

	STA	TTRBA	STA	TTRBA
Range of eye movement (ROEM)	Corneal diameters (CDs)	Corneal diameters (CDs)	*P*	*P*
4-min	Upward-downward 0.39±0.25-0.29±0.31	Upward-downward 0.10±0.12-0.10±0.14	0.0718	0.7188
	Upward-nasal 0.39±0.25-0.22±0.37	Upward-nasal 0.10±0.12-0.10±0.17	**0.0008**	0.4237
	Upward-temporal 0.39±0.25-0.53±0.29	Upward-temporal 0.10±0.12-0.10±0.12	0.0357	0.976
	Downward-nasal 0.29±0.31-0.22±0.37	Downward-nasal 0.10±0.14-0.10±0.17	0.1141	0.6241
	Downward-temporal 0.29±0.31-0.53±0.29	Downward-temporal 0.10±0.14-0.10±0.12	**0.0013**	0.7113
	Nasal-temporal 0.22±0.37-0.53±0.29	Nasal-temporal 0.10±0.17-0.10±0.12	<0.0001	0.3788
10-min	Upward-downward 0.16±0.21-0.15±0.22	Upward-downward 0.06±0.10-0.06±0.11	0.7039	0.992
	Upward-nasal 0.16±0.21-0.06±0.20	Upward-nasal 0.06±0.10-0.07±0.15	**0.0038**	0.5092
	Upward-temporal 0.16±0.21-0.18±0.23	Upward-temporal 0.06±0.10-0.03±0.05	0.7871	0.3788
	Downward-nasal 0.15±0.22-0.06±0.20	Downward-nasal 0.06±0.11-0.07±0.15	0.012	0.5754
	Downward-temporal 0.15±0.22-0.18±0.23	Downward-temporal 0.06±0.11-0.03±0.05	0.5754	0.4122
	Nasal-temporal 0.06±0.20-0.18±0.23	Nasal-temporal 0.07±0.15-0.03±0.05	**0.0054**	0.9362
30-min	Upward-downward 0.10±0.14-0.06±0.09	Upward-downward 0.04±0.10-0.03±0.10	0.3897	0.4715
	Upward-nasal 0.10±0.14-0.01±0.06	Upward-nasal 0.04±0.10-0.05±0.13	0.0142	0.6744
	Upward-temporal 0.10±0.14-0.10±0.16	Upward-temporal 0.04±0.1-0.01±0.03	0.7263	0.2076
	Downward-nasal 0.06±0.09-0.01±0.06	Downward-nasal 0.03±0.10-0.05±0.13	0.075	0.8103
	Downward-temporal 0.06±0.09-0.10±0.16	Downward-temporal 0.03±0.10-0.01±0.03	0.6891	0.6527
	Nasal-temporal 0.01±0.06-0.10±0.16	Nasal-temporal 0.05±0.13-0.01±0.03	0.0601	0.4965

All comparisons were performed using the Mann–Whitney *U* test with Bonferroni correction. *P* value less than 0.0083 was considered statistically significant. STA, sub-Tenon’s capsule anesthesia; TTRBA, trans-Tenon’s capsule retrobulbar anesthesia *P* values in boldface are statistically significant.
